# Supplementary material for: Adsorption of Mono- and Divalent Ions onto Dendritic Polyglycerol Sulfate (dPGS) as Studied Using Isothermal Titration Calorimetry
Source: Polymers (Basel). 2023 Jun 23;15(13):2792. doi: 10.3390/polym15132792 (PMC10346990; doi:10.3390/polym15132792)
Supplement: Supplementary file 1 [file polymers-15-02792-s001.zip › polymers-2461993-supplementary.pdf]

SUPPLEMENTARY MATERIALS

Adsorption of Mono- and Divalent Ions onto Dendritic Polyglycerol Sulfate (dPGS) as Studied Using Isothermal Titration Calorimetry

Jacek J. Walkowiak [1,2,3,*], Rohit Nikam [4] and Matthias Ballauff [5]

[1] DWI—Leibniz-Institute for Interactive Materials e.V, Forckenbeckstraße 50, 52074 Aachen, Germany
[2] Institute of Technical and Macromolecular Chemistry, RWTH Aachen University, Worringerweg 2, 52074 Aachen, Germany
[3] Aachen-Maastricht Institute for Biobased Materials (AMIBM), Maastricht University, Urmonderbaan 22, 6167 RD Geleen, The Netherlands
[4] Helmholtz-Zentrum Berlin für Materialien und Energie, Hahn-Meitner-Platz 1, 14109 Berlin, Germany; rohit.nikam@helmholtz-berlin.de
[5] Institut für Chemie und Biochemie, Freie Universität Berlin, Taktstraße 3, 14195 Berlin, Germany; mballauff@zedat.fu-berlin.de
* Correspondence: walkowiak@dwi.rwth-aachen.de

Single Set of Identical Binding Sites (SSIS) Model.

Subtracting equation (1) into equation (2) gives

$$[A]_{tot} = [A] + \frac{N \cdot K_b[A] \cdot [dPGS]}{1 + K_b[A]} \tag{S1}$$

Solving of equation (S1) for *[A]* leads to a quadratic equation

$$\theta^2 - \theta\left[1 + \frac{[A]_{tot}}{N[dPGS]} + \frac{1}{NK_b[dPGS]}\right] = 0 \tag{S2}$$

The heat Q' after each injection i is equal to

$$Q' = [dPGS]V_0 N\theta\Delta H^{ITC} \tag{S3}$$

Solving the equation (S2) for θ and then substituting this into equation (S3) gives

$$Q' = \frac{N[dPGS]\Delta H^{ITC}V_0}{2}\left[1 + \frac{[A]_{tot}}{N[dPGS]} + \frac{1}{NK_b[dPGS]} - \sqrt{\left(1 + \frac{[A]_{tot}}{N[dPGS]} + \frac{1}{NK_b[dPGS]}\right)^2 - \frac{4[A]_{tot}}{N[dPGS]}}\right] \tag{S4}$$

The analysis includes the effect of the increase of the volume during titration [37,38]. The experimental data are fitted by calculating the heat change of the solution ΔQ_i released with each injection i and corrected for displaced volume ΔV_i ass expressed in equation (4)

ITC Isotherms

a)

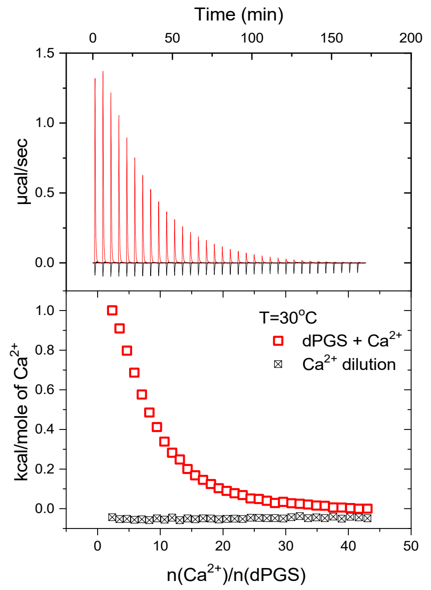

b)

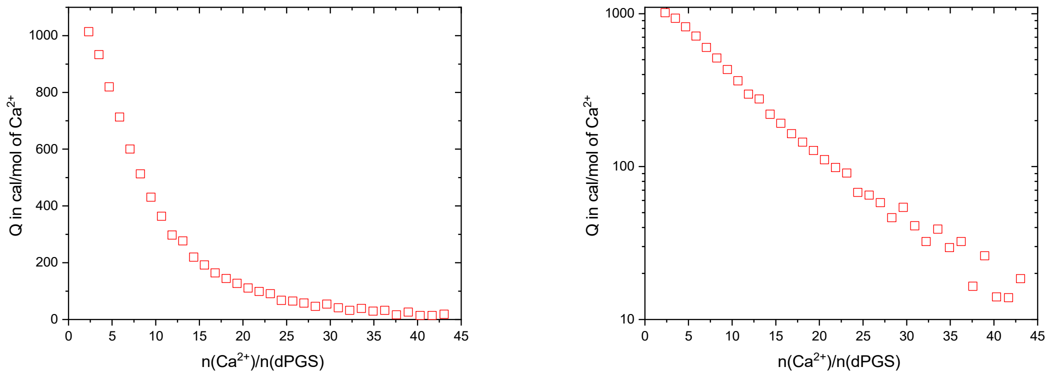

Figure S1. (a) ITC data for the binding of Ca^{2+} ions to dPGS at pH 7.2 and temperature of 30°C in 10 mM MOPS buffer. The upper panel shows the raw data of the binding (red spikes) and the dilution of Mg^{2+} by buffer (black spikes). The integrated heats of each injection are shown in the lower panel. **(b)** Binding isotherms for Ca^{2+} - dPGS interaction, presented on a typical ITC plot (left-handed) and semi-logarithmic plot (right-handed). Resulting $[Ca^{2+}]^{tot}$: 0.8 mM. Plots refer to section 3.1.

a)

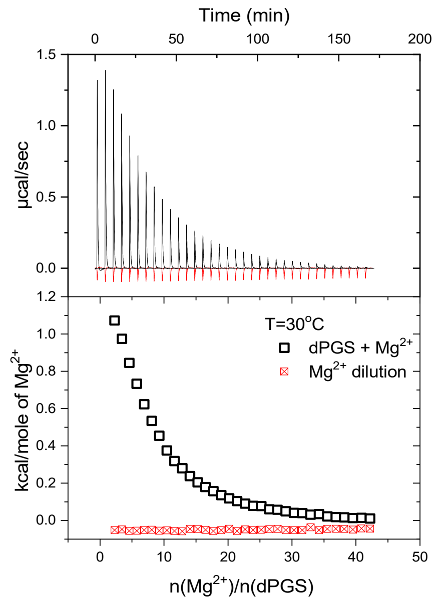

b)

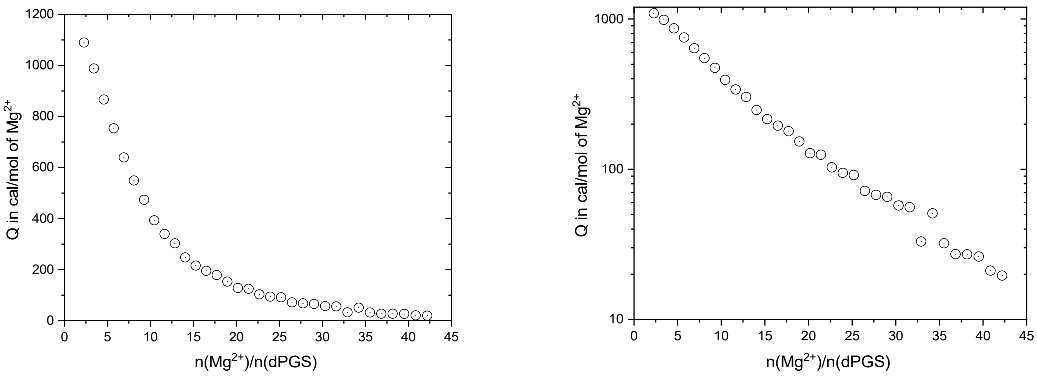

Figure S2. (a) ITC data for the binding of Mg^{2+} ions to dPGS at pH 7.2 and temperature of 30°C in 10 mM MOPS buffer. The upper panel shows the raw data of the binding (black spikes) and the dilution of Mg^{2+} by buffer (red spikes). The integrated heats of each injection are shown in the lower panel. **(b)** Binding isotherms for Mg^{2+} - dPGS interaction, presented on a typical ITC plot (left-handed) and semi-logarithmic plot (right-handed). Resulting $[Mg^{2+}]^{tot}$: 0.8 mM. Plots refer to section 3.1.

a)

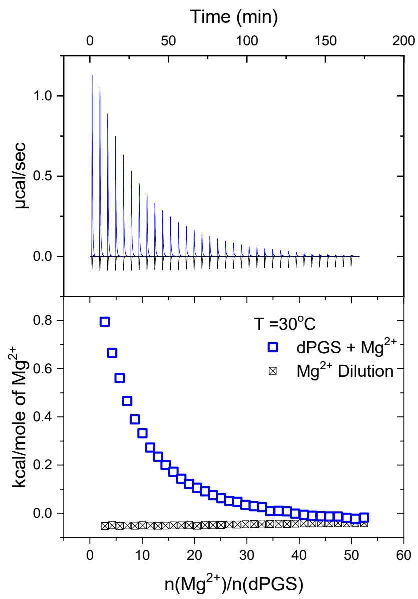

b)

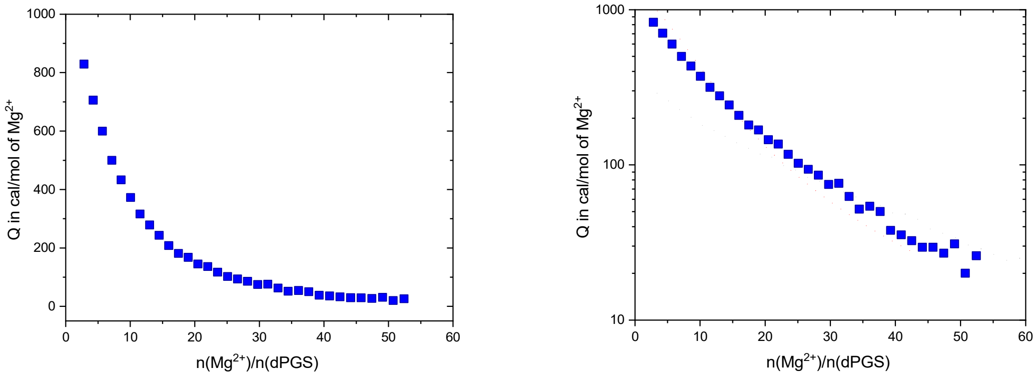

Figure S3. (a) ITC data for the binding of Mg^{2+} ions to dPGS at pH 7.2 and temperature of 30°C in 10 mM MOPS buffer. The upper panel shows the raw data of the binding (blue spikes) and the dilution of Mg^{2+} by buffer (black spikes). The integrated heats of each injection are shown in the lower panel. **(b)** Binding isotherms for Mg^{2+} - dPGS interaction, presented on a typical ITC plot (left-handed) and semi-logarithmic plot (right-handed). Resulting $[Mg^{2+}]^{tot}$: 0.8 mM. Plots refer to section 3.2.

a)

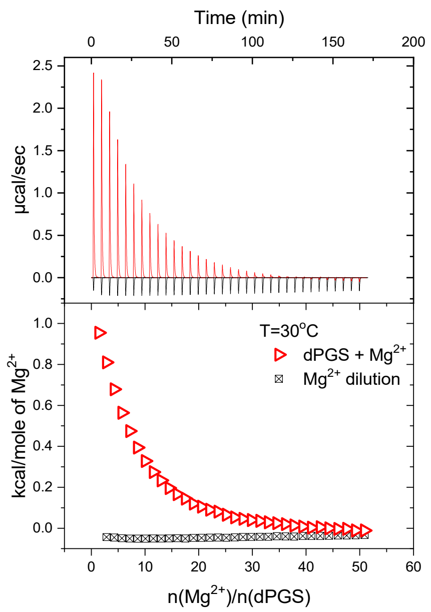

b)

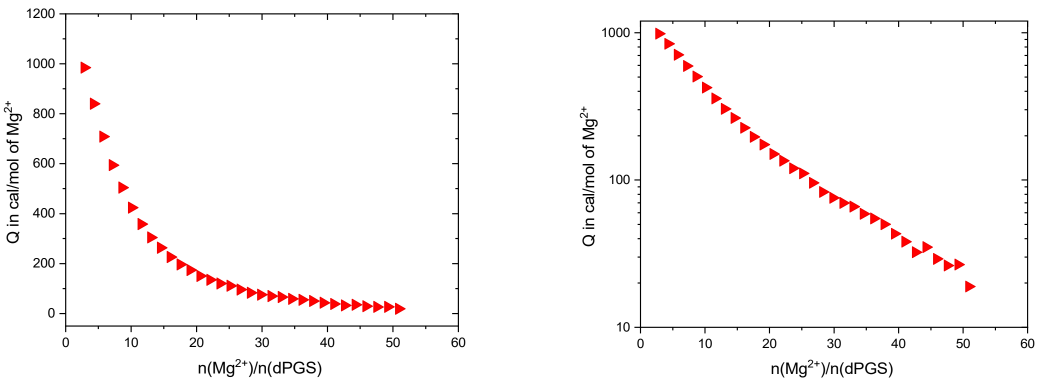

Figure S4. (a) ITC data for the binding of Mg^{2+} ions to dPGS at pH 7.2 and temperature of 30°C in 10 mM MOPS buffer. The upper panel shows the raw data of the binding (red spikes) and the dilution of Mg^{2+} by buffer (black spikes). The integrated heats of each injection are shown in the lower panel. **(b)** Binding isotherms for Mg^{2+} - dPGS interaction, presented on a typical ITC plot (left-handed) and semi-logarithmic plot (right-handed). Resulting $[Mg^{2+}]^{tot}$: 1.6 mM. Plots refer to section 3.2.

a)

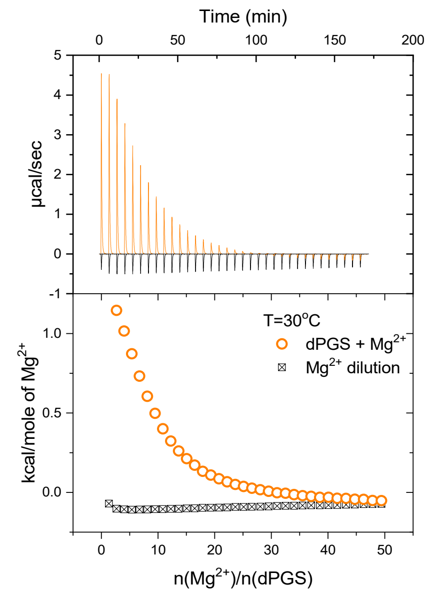

b)

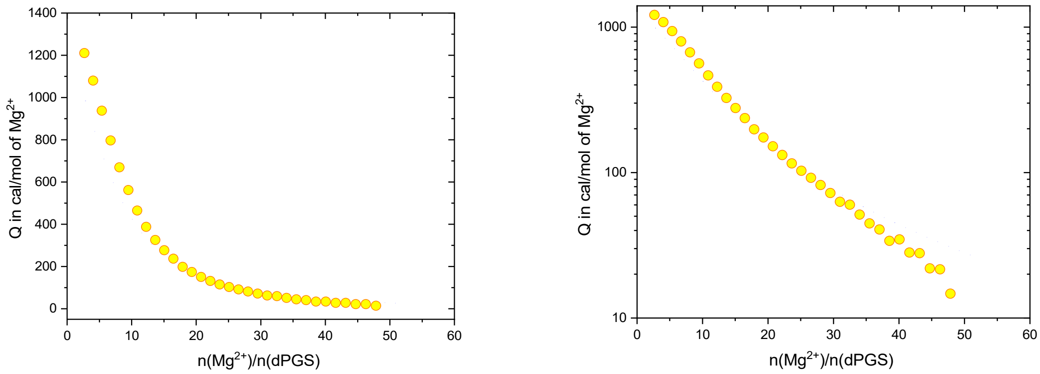

Figure S5. (a) ITC data for the binding of Mg^{2+} ions to dPGS at pH 7.2 and temperature of 30°C in 10 mM MOPS buffer. The upper panel shows the raw data of the binding (yellow spikes) and the dilution of Mg^{2+} by buffer (black spikes). The integrated heats of each injection are shown in the lower panel. **(b)** Binding isotherms for Mg^{2+} - dPGS interaction, presented in a typical ITC plot (left-handed) and semi-logarithmic plot (right-handed). Resulting $[Mg^{2+}]^{tot}$: 2.5 mM. Plots refer to section 3.2.

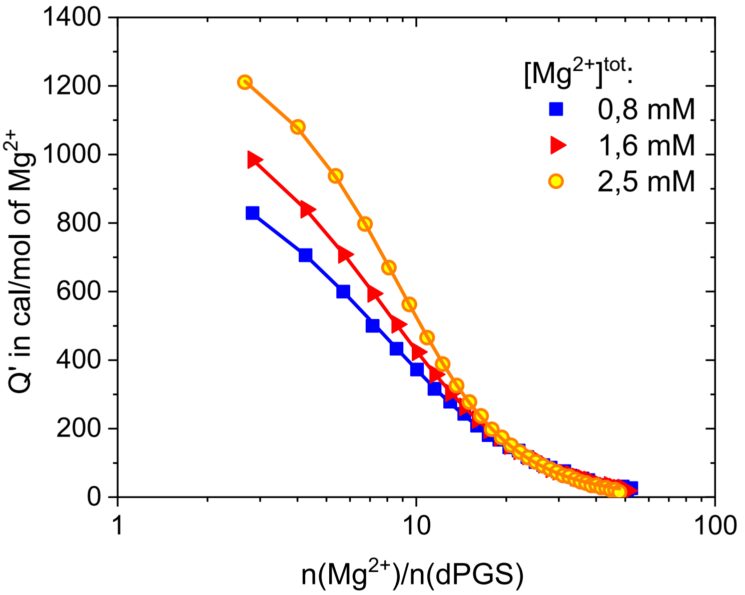

Figure S6. Binding isotherms for Mg^{2+} interacting with dPGS in semi-logarithmic plot. Solid lines represent the SSIS fit. Plot refer to section 3.2.

High ionic strength (I = 31.5 mM)

a)

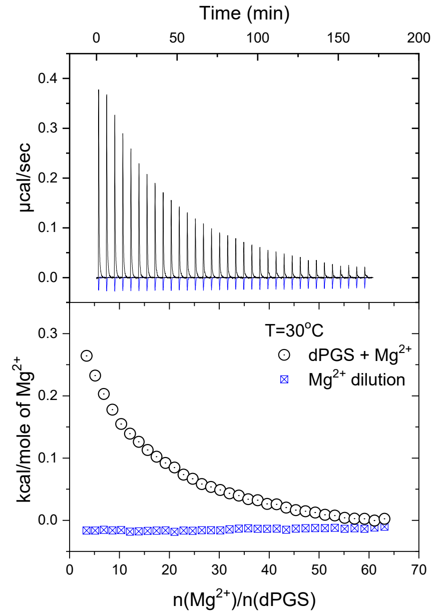

b)

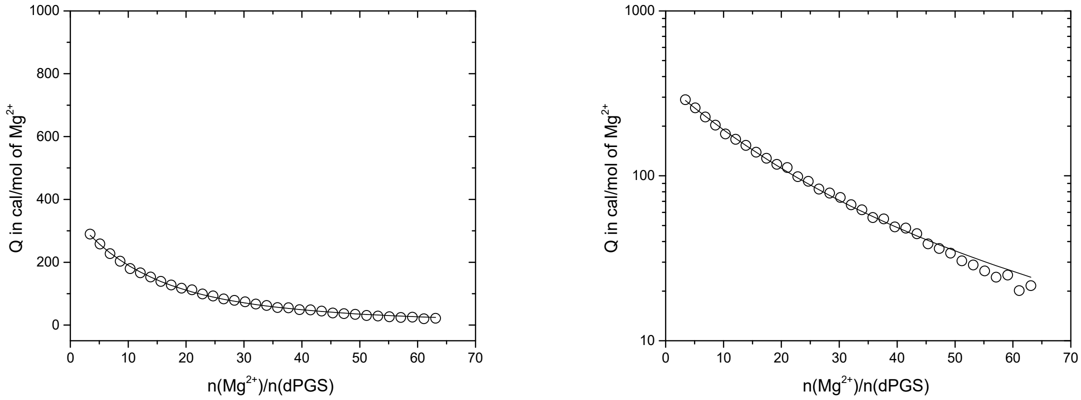

Figure S7. **(a)** ITC data for the binding of Mg^{2+} ions to dPGS at pH 7.2 and temperature of 30°C in 10 mM MOPS buffer. The upper panel shows the raw data of the binding (yellow spikes) and the dilution of Mg^{2+} by buffer (black spikes). The integrated heats of each injection are shown in the lower panel. **(b)** Binding isotherms for Mg^{2+} - dPGS interaction, presented in a typical ITC plot (left-handed) and semi-logarithmic plot (right-handed). Resulting $[Mg^{2+}]^{tot}$: 0.8 mM. Plots refer to section 3.2.

Table S1. Experimental parameters for dPGS-divalent ion measurements (at I = 31.5 mM), conducted on a VP-ITC instrument.

System	Buffer/Ionic strength (mM)	$[DI]^{tot\ a)}$ (mM)	$[Na^+]^{tot}$ (mM)	T [K]	c (DI) $^{b)}$ (mM)	c (dPGS) (mM)
Mg^{2+}/dPGS	MOPS/31.5	0.8	19.1	303	5.0	0.016

[a)] DI – divalent ion, [b)] concentration of divalent ions in the injectant.

References

37. Indyk, L.; Fisher, H.F. [17] Theoretical aspects of isothermal titration calorimetry. In *Methods in Enzymology*; 1998; pp. 350–364.
38. Lin, L.N.; Mason, A.B.; Woodworth, R.C.; Brandts, J.F. Calorimetric studies of the binding of ferric ions to human serum transferrin. *Biochemistry* **1993**, *32*, 9398–9406, doi:10.1021/bi00087a019.
